# Puerarin Attenuates Podocyte Damage in Mice With Diabetic Kidney Disease by Modulating the AMPK/Nrf2 Pathway

**DOI:** 10.1155/ije/4473803

**Published:** 2025-01-21

**Authors:** Song Xue, Wei Fan, Qingping Li, Hong Huang, Yibo Tang, Min Wu

**Affiliations:** ^1^School of Minerals Processing and Bioengineering, Central South University, Changsha, Hunan 410011, China; ^2^Nephrology Department, Jiangxi Provincial Key Research Laboratory of Traditional Chinese Medicine, Key Research Laboratory of Chronic Renal Failure, Affiliated Hospital of Jiangxi University of Traditional Chinese Medicine, Nanchang, Jiangxi 330006, China; ^3^School of Clinical Medicine, Jiangxi University of Traditional Chinese Medicine, Nanchang, Jiangxi 330004, China

**Keywords:** diabetic nephropathy, oxidative stress, podocin, podocyte, puerarin

## Abstract

**Background:** This study aimed to investigate the potential mechanisms of puerarin in alleviating diabetic nephropathy (DKD) in mice.

**Method:** The DKD model was induced by multiple low-dose injections of streptozotocin (STZ) and a high-sugar and high-fat diet in male C57BL/6J mice. After confirming the onset of DKD, mice were given irbesartan, distilled water, or different concentrations of puerarin (40 and 80 mg/kg/d) by gavage for 8 weeks. HE staining and PAS staining were adopted to assess the pathological changes in the kidney tissues. Meanwhile, the levels of superoxide dismutase, catalase, creatinine, and cystatin C in the serum and the urine albumin and creatinine were measured, and the renal indices as well as the urinary albumin-to-creatinine ratio (UACR) were calculated. The changes of podocin and protein expression levels associated with AMPK/Nrf2 signaling pathway were evaluated by western blot.

**Results:** Puerarin significantly reduced the level of fasting blood glucose, renal index, glomerular mesangial expansion index, renal function, and oxidative stress induced by STZ (*p* < 0.05). The pathological injuries in kidney tissues were also alleviated. Furthermore, we demonstrated that the expression level of podocin and protein related to the AMPK/Nrf2 signaling pathway was also decreased significantly by the treatment of puerarin. At the same time, the efficacy of puerarin in the treatment of DKD was better than that of irbesartan, and the treatment effect of the high-dose group (80 mg/kg/d) was also significantly better than that of the low-dose group (40 mg/kg/d).

**Conclusion:** Puerarin could attenuate the severity of DKD and protect the podocyte in mice in a dose-dependent way. Also, it might be performed by regulating the AMPK/Nrf2 pathway. These findings may provide a theoretical basis for updating the clinical management of DKD.

## 1. Introduction

According to the latest Global Diabetes Map (10th edition) published by the International Diabetes Federation (IDF), approximately 537 million adults (aged 20–79 years) worldwide had diabetes by 2021 [1]. This means that diabetes affects 1 in 10 of the world's population. Moreover, it is estimated that the prevalence of diabetes will further increase to 11.3% and 12.2% in 2030 and 2045, respectively [2]. As the most common microvascular complication of diabetes mellitus, diabetic kidney disease (DKD) was the leading cause of end-stage renal disease [3]. It was reported that about 30%–40% of patients with diabetes would develop DKD [4]. Given the rising incidence of diabetes in younger people [5], the occurrence of DKD is anticipated to escalate [6]. However, DKD is still not completely curable, and current treatments are not sufficient to effectively prevent the deterioration of kidney function [7].

In addition, the pathogenesis of DKD remains unclear [8]. Previous studies have found that overactivated oxidative stress plays an important role in the development of DKD [9]. Excess reactive oxygen species (ROS) can cause endothelial cell damage, podocyte atrophy and detachment, morphological and structural changes in the peduncle, and glomerular fibrosis [10]. Therefore, there is a pressing need to develop new drugs that can reduce the level of oxidative stress with minimal damage to normal tissues, and natural herbs documented in Chinese traditional medicine may offer a potential solution.

Puerarin is an isoflavone isolated from the traditional Chinese medicine *Pueraria lobata* [11]. Modern pharmacological studies have demonstrated that puerarin has a variety of pharmacological effects such as anti-inflammatory [12, 13], antioxidant [14, 15], regulation of glucolipid metabolism [16], inhibition of ferroptosis [17], and improvement of mitochondrial function [18]. Studies have shown that puerarin can enhance the activity of antioxidant enzymes and thus protect cells from oxidative stress–induced apoptosis [19]. Moreover, puerarin can activate AMPK phosphorylation to inhibit oxidative stress and improve hepatic mitochondrial respiration to exert a hypoglycemic effect, which may be a potential therapeutic option for the treatment of diabetes mellitus [18]. Recent studies have shown that puerarin can also improve glucose and lipid homeostasis in the liver of obese rats by increasing the phosphorylation levels of AMPK and ACC [20]. In addition, several studies have shown that puerarin protects against neuronal cell damage and inhibits ferroptosis in rats by activating Nrf2 and its downstream pathway pathways [21–23]. However, the potential mechanism of its antioxidative stress effect in DKD with a protective effect on glomerular podocytes remains elusive. Therefore, this study aims to explore the effects and potential mechanisms of puerarin on the level of oxidative stress and podocytes in DKD mice through the AMPK/Nrf2 pathway.

## 2. Materials and Methods

### 2.1. Animals

Fifty 7-week-old male C57BL/6J mice were procured from Hunan Slake Jingda Experimental Animal Co., Ltd. (License Number: SCXK(Xiang)2019-0004). After acclimatization for one week, mice at 8 weeks of old were randomly allocated to the following 5 groups: control group (*n* = 10), DKD group (*n* = 10), DKD + irbesartan (40 mg/kg) group (*n* = 10), DKD + puerarin (40 mg/kg) group (*n* = 10), and DKD + puerarin (80 mg/kg) group (*n* = 10). In compliance with the Association for Animal Models of Diabetic Complications (AMDCC) protocol, diabetes mellitus was induced using multiple intraperitoneal injections of low-dose streptozotocin (STZ) (40 mg/kg for 5 consecutive days) [24]. After fasting for 12 h, mice were injected intraperitoneally with STZ (S0130, Sigma, USA) along with 0.1 mol/L sodium citrate buffer (PHR1416, Sigma, USA) for 5 days, while mice in the control group were administered with isovolumetric sodium citrate buffer (0.1 mol/L). From Day 1 of the start of modeling, all three groups, except the control group, were switched to a high-sugar, high-fat diet. After 4 weeks, the diagnosis of DKD was confirmed by testing mice with random blood glucose levels > 16.7 mmol/L and urinary albumin/creatinine ratio (UACR) ≥ 30 μg/mg [25]. Puerarin used in this study was purchased from Shanghai Aladdin Biochemical Technology Co., Ltd. (China, P111270, purity: ≥ 98%). As for the dosage, concentrations of 40 and 80 mg/kg/d were selected for intervention as reference to the dosage reported in previous study [26] and the pre-experiment in our group.

At 12 weeks of old, the mice in the DKD + irbesartan (40 mg/kg, Y001166, Sigma, USA) group, DKD + puerarin (40 mg/kg) group, and DKD + puerarin (80 mg/kg) group were administered irbesartan (40 mg/kg/d), puerarin (40 mg/kg/d), and puerarin (80 mg/kg/d) by gavage for eight weeks, respectively. Nevertheless, mice in the control group and the DKD group were treated by gavage with equal volume of distilled water. All animal experiments were approved by the Affiliated Hospital of Jiangxi University of Traditional Chinese Medicine (No. 20230730005).

### 2.2. Sample Collection

The level of fasting blood glucose (FBG) and body weight were measured weekly. Also, urine was collected by using metabolic cages (one mouse per cage) from 12-week-old mice. In particular, urine was collected the day after the completion of gavage with puerarin, irbesartan, or distilled water. The collected urine was centrifuged at 4°C, 5000 rpm for 30 min, and the supernatant was stored at −80°C.

Mice were anesthetized and executed using 1% sodium pentobarbital (30 mg/kg) according to universal standards [27]. Subsequently, peripheral blood and bilateral kidney specimens with the renal envelope removed were collected from the mice. The renal index (RI) was then calculated. The peripheral blood was then centrifuged and the supernatant along with the right kidney tissue was stored at −80°C. RI was defined as the ratio of the weight of the kidney (mg) to body weight (g).

### 2.3. Histologic Analysis

All left kidneys were fixed with 4% polyformaldehyde and embedded in paraffin, and sections with a thickness of 3 μm were prepared. After being stained with hematoxylin-eosin (HE) or periodic acid–Schiff (PAS), pathological changes were observed under an optical microscope. We randomly captured 10 high-magnification views containing glomeruli and renal tubular mesenchyme, and the mesangial expansion index (MEI) of the glomeruli was rated with the assistance of ImageJ software. The specific scoring criteria were as follows: (1) normal glomeruli (0 points); (2) the area of intraglomerular mesangial stromal hyperplasia was < 25% (1 point); (3) the hyperplasia area of the mesangial matrix in the glomeruli was between 25% and 50% (2 points); (4) the hyperplasia area of the mesangial matrix in the glomeruli was between 51% and 75% (3 points); and (5) the area of intraglomerular mesangial stromal hyperplasia was > 75% (4 points) [28].

### 2.4. ELISA Analysis

Stored serum and urine were taken out to room temperature. The urine was centrifuged and the supernatant was collected. According to the manufacturer's instructions, the concentrations of the following indicators in the serum were measured, including superoxide dismutase (SOD, A001-3-2, Nanjing Jiancheng Bioengineering Institute), catalase (CAT, A007-1-1, Nanjing Jiancheng Bioengineering Institute), serum creatinine (Scr, E-BC-K188-M, Elabscience Biotechnology Co., Ltd), and serum cystatin C (CysC, E-EL-M3024, Elabscience Biotechnology Co., Ltd). Also, the concentration of albumin (ALB, E-EL-M3032, Elabscience Biotechnology Co., Ltd) and Cr in urine was measured using corresponding ELISA kits, and UACR was counted.

### 2.5. Western Blot Analysis

100 mg of right kidney tissue was lysed in 400 μL of cell lysis buffer on ice, and the supernatant containing total cellular proteins was collected. The BCA assay kit was applied to assess the protein concentration. Proteins were separated using 10% SDS-PAGE and transferred to the PVDF membrane. After being blocked with 5% skim milk at room temperature for 2 h, it was incubated with the following primary antibodies overnight at 4°C (all at a dilution ratio of 1:1000): AMPK (Abcam, ab110036), *p*-AMPK (Abcam, ab92701), Nrf2 (Abcam, ab137550), and podocin (Abcam, ab181143). After incubation with goat anti-mouse secondary antibody (dilution: 1:10,000, Abcam, ab205719) at room temperature for 2 h, images were collected and quantified by ImageJ software. β-Actin (Abcam, ab115777) was used as an internal reference.

### 2.6. Statistical Analysis

SPSS26.0 software was used for statistical analysis. Data with a normal distribution were described by mean ± standard deviation, and comparisons between multiple groups were performed by one-way ANOVA. Data without a normal distribution were described by median (P25 and P75), and comparisons among multiple groups were conducted using the Kruskal–Wallis test. *p* value < 0.05 was considered statistically significant.

## 3. Results

### 3.1. Puerarin Significantly Reduced FBG Level in Mice

**Figure 1:**
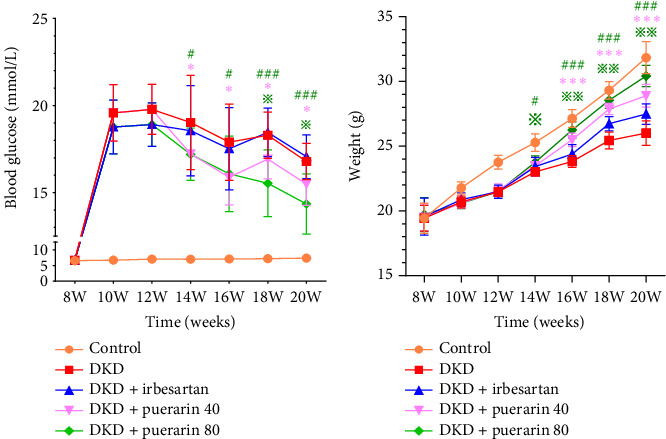
The dynamic monitoring in the level of fasting blood glucose and weight in mice (*n* = 10 for each group). ⁣^∗^*p* < 0.05, ⁣^∗∗^*p* < 0.01, ⁣^∗∗∗^*p* < 0.001, compared with the diabetic kidney disease (DKD) group; ^#^*p* < 0.05, ^##^*p* < 0.01, ^###^*p* < 0.001, compared with the DKD + irbesartan (40 mg/kg) group; ^※^*p* < 0.05, ^※※^*p* < 0.01, compared with the DKD + puerarin (40 mg/kg) group.

Compared with the control group, a significant increase in FBG levels was observed in the DKD group, DKD + irbesartan (40 mg/kg) group, DKD + puerarin (40 mg/kg) group, and DKD + puerarin (80 mg/kg) group (*p* < 0.05). In comparison with the DKD group, both DKD + puerarin (40 mg/kg) and DKD + puerarin (80 mg/kg) groups exhibited a significant reduction in the levels of FBG (*p* < 0.05). Notably, the level of FBG was significantly lower in mice in puerarin (40 and 80 mg/kg) groups compared with DKD + irbesartan (40 mg/kg) group (*p* < 0.05). In addition, a significant difference in FBG levels was observed between the DKD + puerarin (40 mg/kg) group and the DKD + puerarin (80 mg/kg) group (*p* < 0.05, Figure 1).

### 3.2. Puerarin Alleviated the Renal Pathological Injury in a Dose-Dependent Manner

**Figure 2:**
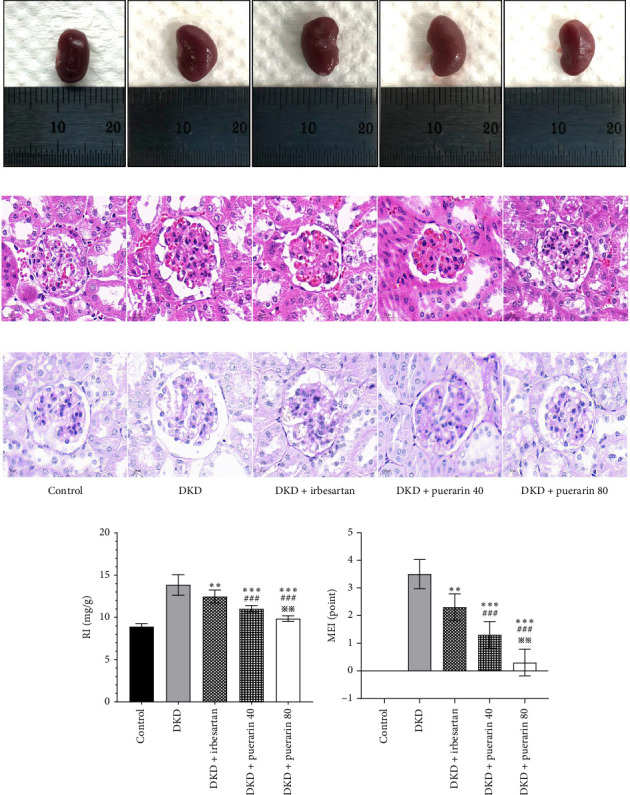
The morphological and histological changes in mice (*n* = 10 for each group). (a) The morphological changes in renal volume. (b) HE staining pictures (1200x magnification). (c) PAS staining pictures (1200x magnification). (d) Changes in renal index (RI). (e) Changes in glomerular mesangial expansion index score. ⁣^∗∗^*p* < 0.01, ⁣^∗∗∗^*p* < 0.001, compared with the diabetic kidney disease (DKD) group; ^###^*p* < 0.001, compared with the DKD + irbesartan (40 mg/kg) group; ^※※^*p* < 0.01, compared with the DKD + puerarin (40 mg/kg) group.

After STZ modeling, the nephropathy of mice was shown by a significant increase in renal volume in the other four groups except for the control group (Figure 2(a)). We also found that mice in the DKD group presented classic pathological changes related to the DKD, which include significant hypertrophied glomeruli, noticeable mesangial matrix proliferation, increased thickness of the capillary basement membrane, and sharp inflammatory cell infiltration in the renal tubulointerstitial area. The administration of irbesartan and gelsemium effectively mitigated all the aforementioned pathologic changes. In comparison to the DKD + irbesartan (40 mg/kg) group, the inflammatory cell infiltration within the renal tubulointerstitial area was significantly reduced in the puerarin groups. Also, there was a significant difference in the inflammatory cell infiltration within the renal tubulointerstitial area between the DKD + puerarin (40 mg/kg) group and DKD + puerarin (80 mg/kg) group (Figures 2(b) and 2(c)).

The RI levels of DKD group, DKD + irbesartan (40 mg/kg) group, and puerarin group were significantly higher than those in the control group (*p* < 0.05). The irbesartan and puerarin groups significantly reduced the RI of DKD mice (*p* < 0.01). Moreover, there was a significant difference in the DKD + irbesartan (40 mg/kg) group and the puerarin groups regarding the RI (*p* < 0.001). Notably, the effect of reducing RI was more significant in the DKD + puerarin (80 mg/kg) group than in the DKD + puerarin (40 mg/kg) group (*p* < 0.01, Figure 2(d)).

The scores from glomerular MEI manifested that there was a significant decrease in the glomerular MEI in the puerarin groups compared to the DKD group (*p* < 0.001). There was also a significant drop in glomerular MEI between the DKD + puerarin (80 mg/kg) and the DKD + puerarin (40 mg/kg) groups (*p* < 0.01, Figure 2(e)).

### 3.3. Puerarin Is Significantly Superior to Irbesartan in Improving Kidney Function

**Figure 3:**
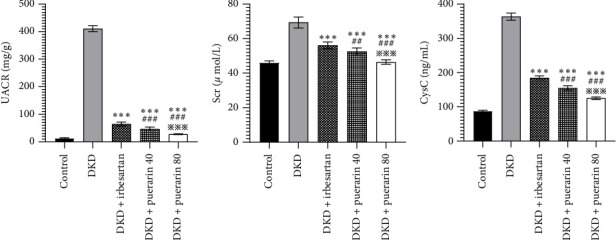
The changes in the level of UACR, Scr, and CysC in mice (*n* = 10 for each group). ⁣^∗∗∗^*p* < 0.001, compared with the diabetic kidney disease (DKD) group; ^##^*p* < 0.01, ^###^*p* < 0.001, compared with the DKD + irbesartan (40 mg/kg) group; ^※※※^*p* < 0.001, compared with the DKD + puerarin (40 mg/kg) group.

The level of UACR, Scr, and CysC rose significantly in the DKD group, DKD + irbesartan (40 mg/kg) group, and puerarin groups (*p* < 0.05), compared to the control group. Moreover, the level of UACR, Scr, and CysC descended significantly in the DKD + irbesartan (40 mg/kg) group and puerarin groups when compared to the DKD group (*p* < 0.001). Besides, the level of UACR, Scr, and CysC was significantly reduced in puerarin groups in comparison with the DKD + irbesartan (40 mg/kg) group (*p* < 0.001). Also, there was a significant difference between the DKD + puerarin (40 mg/kg) group and the DKD + puerarin (80 mg/kg) group concerning the level of three indicators mentioned above (*p* < 0.001, Figures 3(a), 3(b), and 3(c)).

### 3.4. Puerarin Works by Improving Levels of Oxidative Stress in the Kidneys

**Figure 4:**
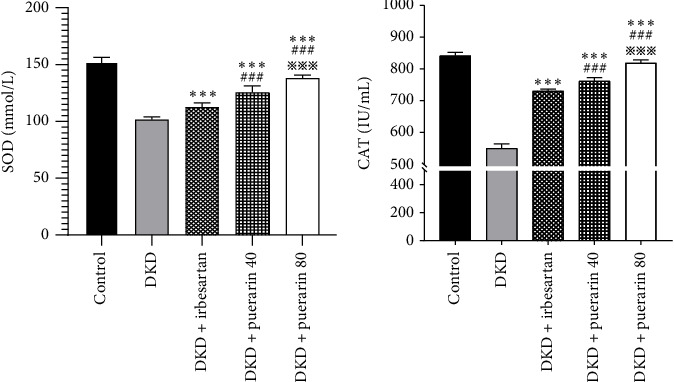
The changes in the level of superoxide dismutase (SOD) and catalase (CAT) in mice (*n* = 10 for each group). ⁣^∗∗∗^*p* < 0.001, compared with the diabetic kidney disease (DKD) group; ^###^*p* < 0.001, compared with the DKD + irbesartan (40 mg/kg) group; ^※※※^*p* < 0.001 compared with the DKD + puerarin (40 mg/kg) group.

Compared to the control group, the level of SOD and CAT in the DKD group, DKD + irbesartan (40 mg/kg) group, and puerarin groups significantly ascended (*p* < 0.05). Besides, the levels of SOD and CAT in the DKD + irbesartan (40 mg/kg) group and puerarin groups significantly decreased than those in the DKD group (*p* < 0.001). Furthermore, the level of SOD and CAT in the puerarin groups significantly descended when compared to the DKD + irbesartan (40 mg/kg) group (*p* < 0.001). Moreover, a significant difference was observed between the DKD + puerarin (40 mg/kg) group and the DKD + puerarin (80 mg/kg) group in terms of the levels of the two mentioned indicators (*p* < 0.001, Figures 4(a) and 4(b)).

### 3.5. Puerarin May Modulate Oxidative Stress by Increasing the Expression of *p*-AMPK, Nrf2, and Podocin Proteins

**Figure 5:**
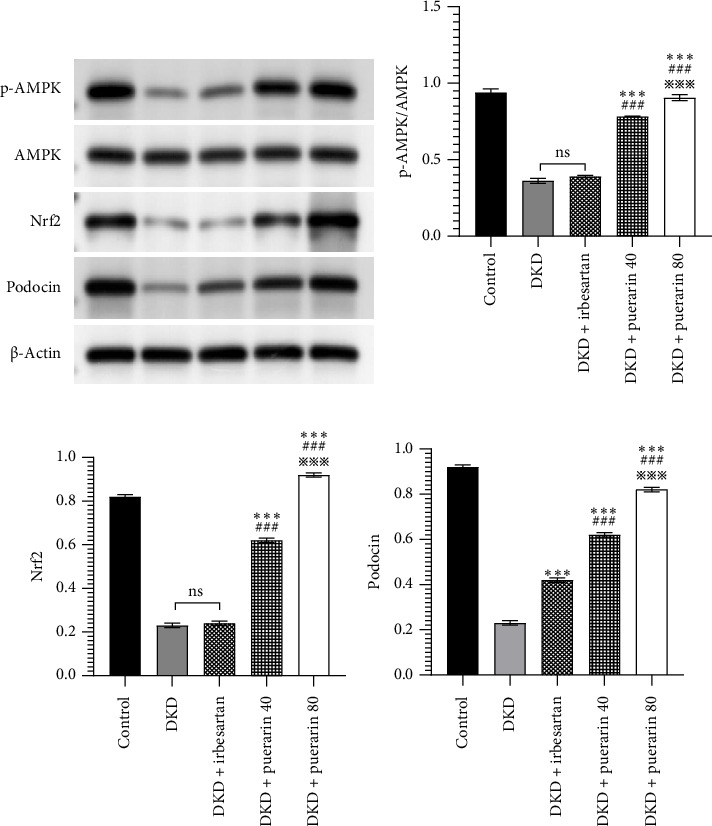
The changes in the expression level of protein in AMPK/Nrf2 pathway and podocin in mice (*n* = 10 for each group). (a) Renal tissue protein blot analysis results. (b) p-AMPK/AMPK results. (c) Nrf2 results. (d) Podocin protein results. ⁣^∗∗∗^*p* < 0.001, compared with the diabetic kidney disease (DKD) group; ^###^*p* < 0.001, compared with the DKD + irbesartan (40 mg/kg) group; ^※※※^*p* < 0.001 compared with the DKD + puerarin (40 mg/kg) group.

The expression of *p*-AMPK, Nrf2, and podocin proteins was shown to be associated with oxidative stress, so we hypothesized that puerarin may reduce oxidative stress and thus ameliorate DKD through the above pathways. The results showed that the protein expressions of *p*-AMPK and Nrf2 in the DKD group were significantly downregulated compared with the control group, and the *p*-AMPK/AMPK ratio was also significantly reduced (*p* < 0.05). However, the use of puerarin could significantly increase the expression level of *p*-AMPK and Nrf2 proteins (*p* < 0.001). Moreover, there was a significant difference between the DKD + puerarin (40 mg/kg) group and the DKD + puerarin (80 mg/kg) group (*p* < 0.001, Figures 5(a), 5(b), and 5(c)).

In addition, irbesartan and puerarin significantly increased the expression of podocin protein in renal tissue than that in the DKD group (*p* < 0.001). We also found that puerarin had the largest increase in podocin protein expression in the 80 mg/kg/d group, which was higher than that in the DKD + irbesartan (40 mg/kg) group and puerarin (40 mg/kg) group (*p* < 0.001, Figures 5(a) and 5(d)).

## 4. Discussion

DKD, as one of the most severe complications of diabetes, was characterized by the persistent increase in urinary protein excretion and progressive decline in renal function [29]. Previous studies have confirmed that oxidative stress could cause most of the pathological changes in DKD and therefore played a key role in the onset and development of DKD. Natural medicinal herbs with low toxicity documented in traditional Chinese medicine had certain advantages for DKD management because they could reduce proteinuria, postpone the deterioration of renal function, and avoid the risk of the occurrence of hypotension [30]. However, due to the complex composition of traditional Chinese medicine and the scarcity of corresponding pharmacological research, their application was limited. Therefore, it is of great importance to discover and validate the primary active ingredients of medicinal herbs and their potential benefits for DKD management, which would contribute to improving the prognosis of patients with DKD.

Lobed kudzuvine root has been adopted to treat diabetes since ancient times. Puerarin, as the main active ingredient of lobed kudzuvine root, has various pharmacological effects such as alleviating hypertension and hyperglycemia, mitigating insulin resistance and antioxidative stress, protecting myocardial cells, improving circulation, enhancing immunity, and inhibiting the renin-angiotensin system [31–35].

In this study, STZ was adopted to induce type 1 diabetes in mice, in which DKD was developed. The pathological features were glomerular hypertrophy, mesangial matrix hyperplasia, increased thickness of capillary basement membrane, and inflammatory cell infiltration in the renal tubulointerstitial area [36]. The histological changes in the DKD group were completely consistent with the pathological changes described above. Moreover, the level of proteinuria and abnormal value for indicators of renal function (UACR, Scr, and CysC) significantly increased. All the changes (including morphology, histology, and biomarkers) in the DKD group have confirmed the onset of DKD.

SOD, as an antioxidant metalloenzyme, could eliminate excessively accumulated free radicals. Therefore, it played a crucial role in the balance of oxidation and antioxidation. CAT belongs to the antioxidant enzyme system. The excess oxygen radicals were firstly decomposed by SOD to generate oxidant-toxic hydrogen peroxide (H_2_O_2_) and oxygen (O_2_), which were further separated into water and oxygen, and therefore damage caused by H_2_O_2_ could be avoided [37]. In the present study, the administration of puerarin could reduce the level of SOD and CAT significantly than those in the DKD group and DKD + irbesartan (40 mg/kg) group, suggesting that puerarin might perform the kidney-protective effect by decreasing the level of oxidative stress in mice, and the effect the puerarin performed was stronger than that of irbesartan.

Furthermore, we investigated the effect of puerarin on the AMPK/Nrf2 signal pathway and the expression level of podocin. AMPK expressed in the kidney was the main intracellular ROS sensor [38], which acted upstream of the Nrf2 antioxidant response element (ARE) pathway and plays a key role in the antioxidant defense mechanism [39]. Consistent with the previous studies, the expression level of *p*-AMPK and Nrf2 proteins showed a significant reduction in the mice with DKD, indicating that the AMPK/Nrf2 signaling pathway in DKD was inhibited [40]. However, the administration of high-concentration puerarin could significantly increase the expression levels of *p*-AMPK and Nrf2 proteins. Our findings indicated that puerarin could reverse the activity of the AMPK/Nrf2 pathway, which was performed by increasing the phosphorylation level of AMPK, promoting the nuclear translocation of Nrf2, and thereby affecting the levels of downstream oxidative stress factors.

Podocytes are highly differentiated epithelial cells, which are essential for the formation and maintenance of the glomerular filtration barrier [41]. Changes in the structure and function of podocytes resulted in proteinuria in DKD and contributed to the continuous development of DKD [42]. Podocin, on the podocyte slit diaphragm, was the key to maintaining the structure stability and normal function of the podocyte, which could prevent the large molecules from passing the glomerular filtration membrane [43]. Our findings show that puerarin could upregulate the expression level of podocin in renal tissue, along with a decrease in pathological damage and a reduction in the level of proteinuria in mice. Puerarin may protect podocytes by increasing the expression level of podocin protein in the kidney and further cause a reduction in proteinuria. However, our research also has some shortcomings. We focused more on the protective effect of puerarin on the kidneys, but lacked monitoring of mouse serum insulin and blood pressure. In future studies, we will further determine whether puerarin indirectly protects the kidneys by improving insulin and blood pressure or directly affects the kidneys.

In conclusion, puerarin can mitigate the damage of podocytes in mice with DKD. The underlying mechanism of the kidney-protective effect may be performed by the upregulation of the AMPK/Nrf2 signaling pathway and the reduction of oxidative stress levels in mice with DKD.

## 5. Conclusion

Puerarin could attenuate the severity of DKD and protect the podocyte in mice in a dose-dependent way. Also, it might be performed by regulating the AMPK/Nrf2 pathway. The choice of dose and duration of treatment with puerarin in this study was based on the published literature. In the future, we will further clarify the concentration and time point of puerarin in the treatment of DKD. Meanwhile, the specific molecular mechanism of puerarin in treating DKD will be further verified in in vitro experiments on podocytes. This study may provide a new theoretical basis for the clinical management of DKD.

## Data Availability

The data used to support the study are available from the corresponding author.

## Nomenclature

DKD: Diabetic kidney disease
UACR: Urinary albumin/creatinine ratio
HE: Hematoxylin-eosin
PAS: Periodic acid–Schiff
RI: Renal index
SOD: Superoxide dismutase
CAT: Catalase
Scr: Serum creatinine
CysC: Cystatin C
ALB: Albumin
MEI: Mesangial expansion index
